# Feature importance guided autoencoder for dimensionality reduction in intrusion detection systems

**DOI:** 10.1038/s41598-026-36695-9

**Published:** 2026-02-04

**Authors:** Mohamed A. Abdel-Rahman, Ala Saleh Alluhaidan, Sahar A. El-Rahman, Ahmed E. Masnour, Ahmed S. I. Amar, Mohamed A. Sobh, Ayman M. Bahaa-Eldin, Tamer Shamseldin, Mohamed Shalaby

**Affiliations:** 1 Department of Computer Engineering and Systems, Ain Shams University, Cairo, Egypt; 2 Department of Information Systems, College of Computer and Information Science, Princess Nourah Bint Abdulrahman University, Riyadh, Saudi Arabia; 3 Computer Systems program - Electrical Engineering Department, Faculty of Engineering - Shoubra, Benha University, Cairo, Egypt; 4 Faculty of Computer Science, Misr International University, Cairo, Egypt; 5 Egyptian Technical Research and Development Center, Cairo, Egypt; 6 Technical Research Center, Cairo, Egypt

**Keywords:** Network security, Intrusion detection system, Dimensionality reduction, Autoencoder, Feature importance, Random forest, Computer science, Information technology

## Abstract

Intrusion detection systems (IDS) play a vital role in protecting computer networks from malicious activities. Dimensionality reduction techniques are commonly employed to enhance the effectiveness and accuracy of machine learning based IDS. In this study, we proposed an effective dimensionality reduction technique called feature importance-based autoencoder (FI-AE) for intrusion detection systems. Our proposed approach encompasses several key components. First, we introduce a novel feature importance method known as one-versus-all feature importance (OVA), which utilizes a random forest algorithm. Next, we train an autoencoder model using a weighted loss function that takes into account the feature importance values obtained through the OVA method. Finally, we utilized the trained autoencoder to reduce the number of features in the benchmark datasets, followed by the application of a random forest classifier to the reduced datasets. We tested our proposed model using three well-known datasets, namely NSL-KDD, UNSW-NB15, and CIC-IDS2017. The experiments revealed that the random forest classifier, combined with our proposed model, outperformed previous dimensionality reduction techniques in terms of accuracy and F1-score.

## Introduction

An intrusion detection system (IDS) is a hardware or software program that actively monitors a network to detect and identify any unauthorized or malicious activities, as well as any violations of established policies. It acts as an adaptable safeguard technology for system security after traditional technologies fail^1^. Cyberattacks are increasing rapidly and becoming more sophisticated, so it is important that protection technologies adapt along with their threats^2^. Machine learning and deep learning models are commonly utilized to enhance the performance of the IDS^3^. The effectiveness of these models heavily relies on the quality and relevance of the data used for training. With the increasing complexity and dimensionality of network traffic data, traditional feature selection methods may struggle to capture the most discriminative information, leading to reduced detection accuracy and increased computational overhead^4^.

Dimensionality reduction techniques have been widely employed in intrusion detection systems to improve the performance and efficiency of anomaly detection algorithms. Principal Component Analysis (PCA) and Autoencoders (AE) are often employed as dimensionality reduction methods^5^. Nevertheless, while these techniques may yield satisfactory results, the task of dimensionality reduction and extracting the most relevant features remains challenging, especially when dealing with highly dimensional and unbalanced datasets. To tackle these issues, several researchers have suggested altered autoencoder structures, such as stacked autoencoders and variational autoencoders^6,7^. These approaches aim to enhance the reconstruction performance and capture more intricate patterns in the data. However, they still do not explicitly address the issue of feature importance and the imbalanced nature of intrusion detection datasets while constructing the new features.

Existing dimensionality reduction techniques for IDS typically optimize reconstruction error or variance capture without explicitly accounting for how features contribute to individual attack classes. This can bias learned representations toward majority classes and reduce sensitivity to minority but critical attack types. Our work is motivated by the need for a reduction method that explicitly incorporates per-class feature relevance to improve detection of minority/rare attacks and to obtain compact representations that preserve attack-discriminative information.

The main contributions and the novelties of this paper are:

1. We propose One-Versus-All (OVA) Feature Importance, a novel scheme that computes feature importance per class (using a binary RF per class) and aggregates these importance scores into an overall weight vector that emphasizes features relevant to at least one class. The choice of using RF is based on a comparison that has been made between machine and deep learning models, namely RF, XGB, KNN, DT, SVM, DNN, CNN, LSTM, CNN-LSTM, TNN. The results show that Both random forest and XGB achieved high accuracy, however RF has less training time.
2. We introduce a weighted mean squared error (WMSE) training objective that integrates OVA importance as per-feature weights in the autoencoder reconstruction loss, focusing representation learning on attack-discriminative features.
3. We design and evaluate the Feature Importance-based Autoencoder (FI-AE) and show how it can be used to produce compact feature sets (16-dimensional bottleneck in our study) that improve downstream classification with an off-the-shelf Random Forest.
4. We provide additional methodological clarifications, variable definitions, and practical recommendations to reduce computational cost and improve reproducibility (e.g., training-time mitigation strategies).

The remainder of this paper is organized as follows. The section “Related work” surveys recent work and positions our approach. The section “Proposed work” details the proposed OVA importance, WMSE, and FI-AE model. The section “Experiments and evaluation” describes experiments, preprocessing, and evaluation; it also discusses practical computational considerations and limitations. The section “Conclusion and future work” concludes and outlines future work.

## Related work

Previous work on dimensionality reduction for intrusion detection systems has mostly focused on feature selection and feature extraction^8^. Feature selection involves identifying the most relevant features using suitable feature importance algorithms, which assess the relevance of each feature based on certain criteria^9^. Feature extraction or feature projection, on the other hand, aims to transform the original features into a lower-dimensional representation while preserving essential information, such as principal component analysis and autoencoders^10^.

PCA is a linear transformation approach that attempts to identify a group of uncorrelated variables known as principal components that represent the greatest amount of variation in the data^11^. Because of its simplicity and efficacy, it has been frequently used for extracting features in IDS. In contrast, autoencoders are neural network-based models that learn to rebuild input data by encoding it into a lower-dimensional form and then decoding it back to the original input space^12^. Autoencoders have demonstrated encouraging results in a variety of applications, including intrusion detection^13^.

Several representative approaches are summarized below, and a comparative table (method, core idea, datasets used, limitations) is provided after the methodological description to improve readers’ ability to compare related work (Reviewer 2 request).

*Hybrid filter-wrapper and ensemble methods.* IG-PCA^14^, ensemble approaches^15,16^, and filter-ensemble methods^17^ have shown that combining multiple selection strategies often improves robustness. Some recent studies also explored meta-heuristics (e.g., Salp Swarm^18^) and evolutionary wrappers^7^ for aggressive dimensionality reduction.

*Autoencoder and deep feature extraction.* Stacked autoencoders, variational autoencoders, and sparse architectures have been applied for IDS feature extraction^6,7^. Recent deep extractors (e.g., DOC-IDS, DOC-like architectures^19^) aim to learn features that maximize anomaly separation.

*Feature-importance informed reduction.* Methods that combine supervised importance (e.g., XGBoost) with dimensionality reduction were proposed in^20,21^. These methods motivate our approach: rather than using a single global importance score, compute per-class importance to avoid majority-class bias.

Table 1 summarizes the related work mentioned in this section.

**Table 1:** Selected related works: method, datasets, and main limitations.

Method (ref)	Datasets	Key limitation
IG-PCA^14^	NSL-KDD, others	May lose class-specific discriminative features when PCA dominates
XGBoost + RNN^20^	NSL-KDD, UNSW-NB15	Relies on single importance measure; may bias to majority classes
AE-PCA fusion^22^	CIC-IDS2017	Complexity and potentially large training requirement
Salp Swarm + Kalman^18^	NSL-KDD, CIC-IDS2017	Heuristic nature may not generalize across datasets
DOC-IDS^19^	CIC-IDS2017	Deep models can be expensive to train and require careful tuning

## Proposed work

In this section, we introduce our proposed autoencoder model for dimensionality reduction in the context of intrusion detection systems. Our initial task involves creating a new approach for determining feature importance based on random forest feature importance called one-versus-all feature importance. Furthermore, a weighted mean square error (WMSE) loss function is included for the purpose of training the autoencoder. Finally, we propose an autoencoder model using the WMSE loss function.

### Random forest feature importance

**Fig. 1:**
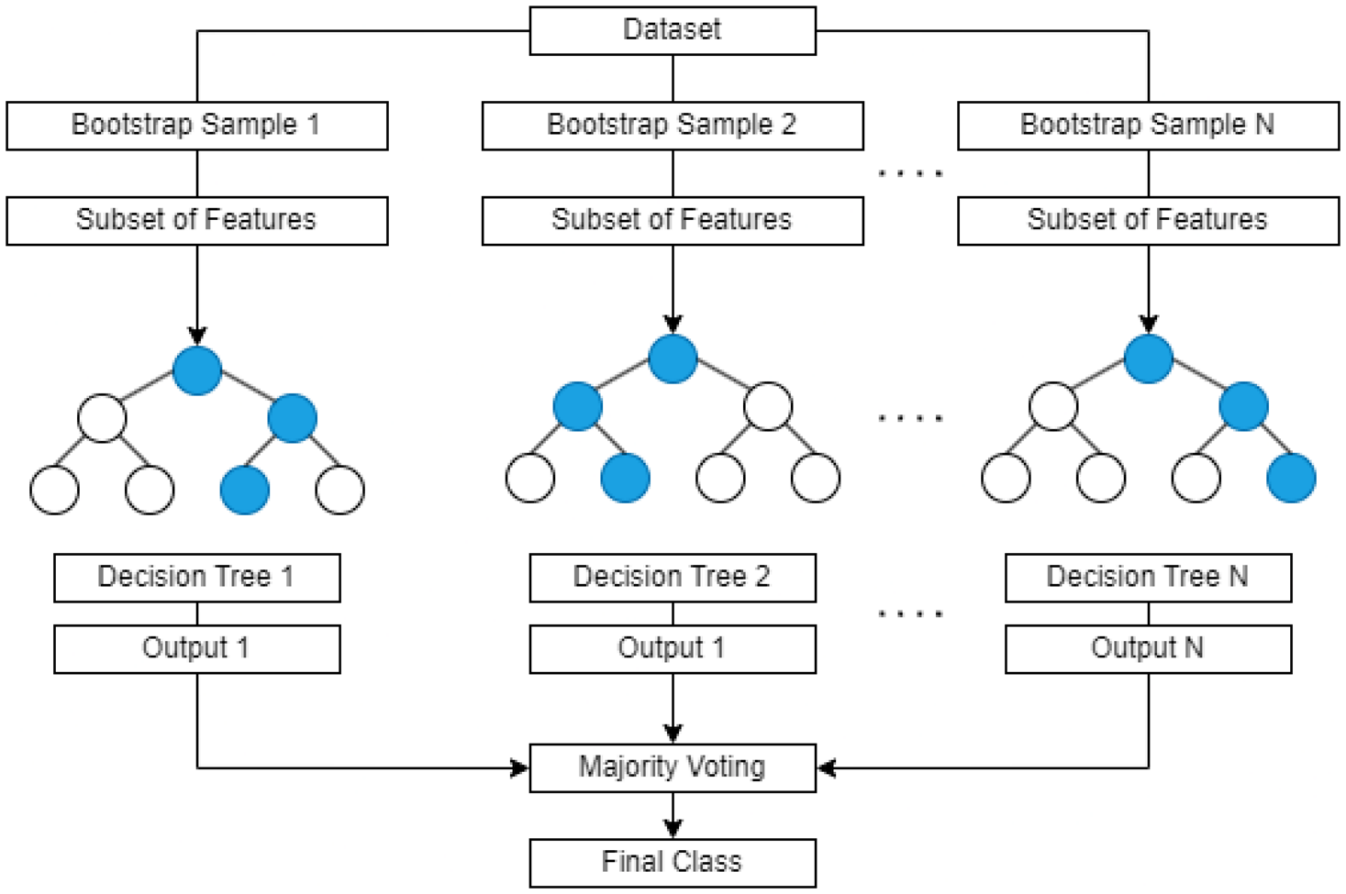
Random forest classifier.

Random forest is an ensemble learning technique that amalgamates multiple decision trees to enhance the accuracy and resilience of predictions. It is a widely favored algorithm in the domain of machine learning, extensively utilized for tasks involving classification and regression. The functioning of random forest involves the random selection of a subset of training samples with replacement to form a bootstrap sample, followed by the random selection of a subset of features from the complete feature set, a process referred to as feature randomness or feature bagging as illustrated in Fig. 1. Subsequently, a decision tree is constructed utilizing the bootstrap sample and the chosen features. Each decision tree within the ensemble contributes to the prediction of the output for a given input, and the final output is derived by amalgamating the predictions from all decision trees^23^.

Random forest can be used as a method for selecting features, where it provides relevance ratings for each feature. The feature importance in random forests is typically calculated as the decrease in node impurity weighted by the probability of reaching that node. The node impurity can be calculated using the Gini Index or the entropy as shown in Eqs. 1 and 2, where $p_i$ is the probability of the class *i* and *c* is the number of classes.

1 $$\begin{aligned} \text {Gini Index}= & 1 - \sum _{i=1}^{c}{p_i^2} \end{aligned}$$

2 $$\begin{aligned} \text {Entropy}= & -\sum _{i=1}^{c}{p_i\log _2 p_i} \end{aligned}$$

A lower Gini index or entropy signifies decreased impurity and demonstrates that splits lead to more homogeneous nodes^24^. The node probability can be calculated by the number of samples that reach the node, divided by the total number of samples.

3 $$\begin{aligned} n_j = w_j C_j - w_{\text {left}(j)} C_{\text {left}(j)} - w_{\text {right}(j)} C_{\text {right}(j)} \end{aligned}$$

Equation 3 illustrates how to calculate the node importance $n_j$, where $w_j$ is the weighted number of samples reaching node *j* and $C_j$ the impurity value of node *j*^25^.

The importance for each feature $f_i$ on a decision tree is calculated as

4 $$\begin{aligned} f_i = \frac{\sum _{j=1}^{l} n_j}{\sum _{k=1}^{m} n_k} \end{aligned}$$

where *l* is the number of nodes for a specific feature *i*, and *m* is the total number of nodes on a decision tree. The final feature importance is determined by summing the feature importance value for each tree and then dividing by the total number of trees.

### One-versus-all feature importance

As previously explained, the feature importance for each node in random forests is weighted by the probability of reaching that node. This causes the feature importance to be skewed towards the majority classes, resulting in the model assigning greater importance to the features necessary for classifying majority classes and lesser importance to those needed for classifying the minority class. This bias occurs because the model tends to focus more on correctly classifying the majority class due to its prevalence in the data. However, this bias is not inherent to the feature importance calculation itself, but rather reflects the model’s behavior in the context of imbalanced data. To address this, we propose a new approach for determining feature importance, referred to as one-versus-all (OVA) feature importance. The aim of this method is to alleviate the bias of random forest feature importance towards majority classes, which often leads to inadequate feature importance for minority classes.

In order to mitigate this bias, we compute the feature importance for each class separately by setting the label of the class to 1 and others to 0, and then utilize random forest to determine the feature importance of that specific class. Following this, we standardize the importance values for each class by dividing them by the highest importance value for that class. As a result we get $F_{m \times n}$ matrix, where *m* is the total number of features and *n* is the number of classes.

5 $$\begin{aligned} F_{m \times n} = \begin{bmatrix} f_{11} & f_{12} & f_{13} & \dots & f_{1n} \\ f_{21} & f_{22} & f_{23} & \dots & f_{2n} \\ \vdots & \vdots & \vdots & \ddots & \vdots \\ f_{m1} & f_{m2} & f_{m3} & \dots & f_{mn} \end{bmatrix} \end{aligned}$$

**Fig. 2:**
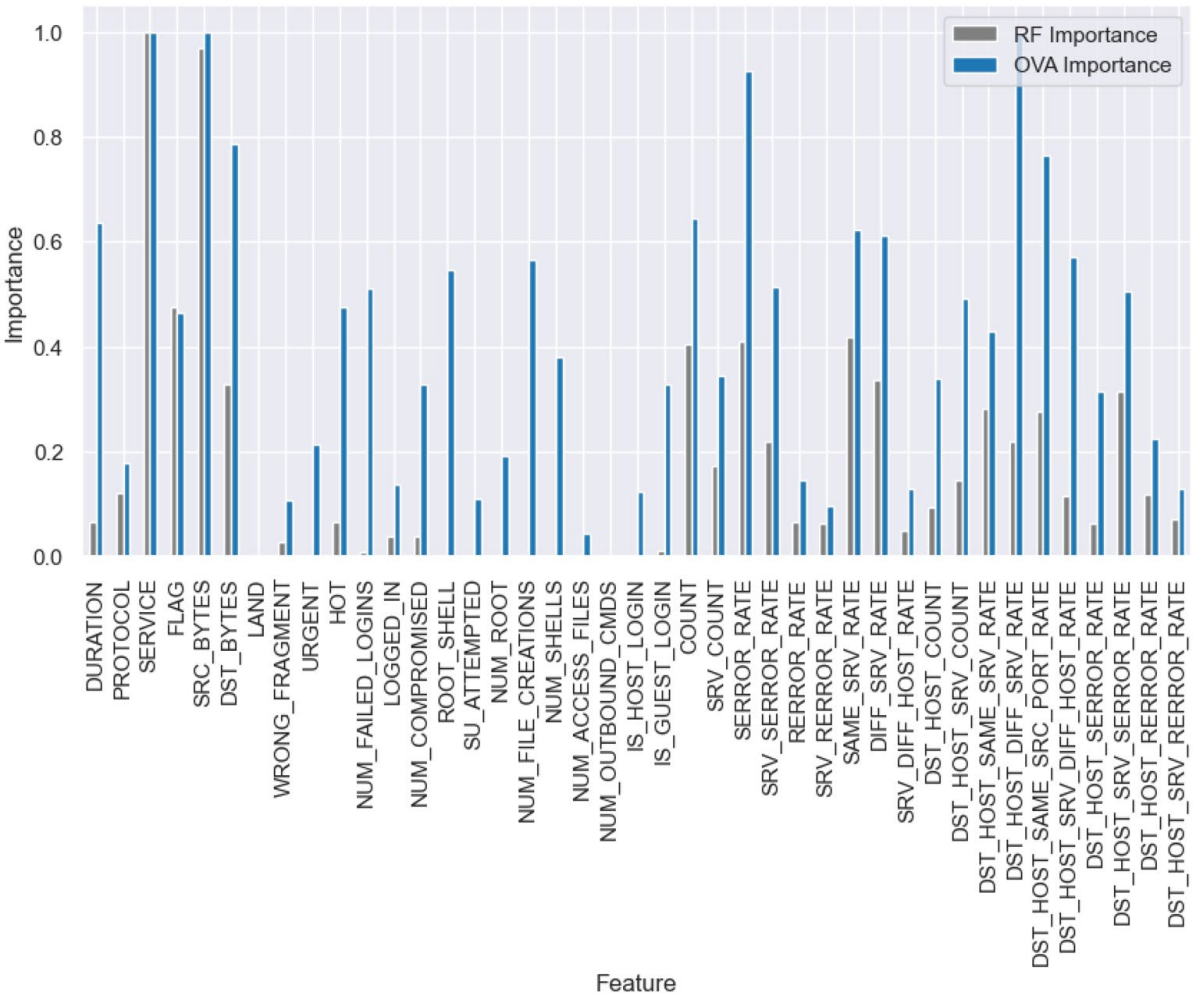
NSL-KDD dataset feature importance (RF vs OVA).

**Fig. 3:**
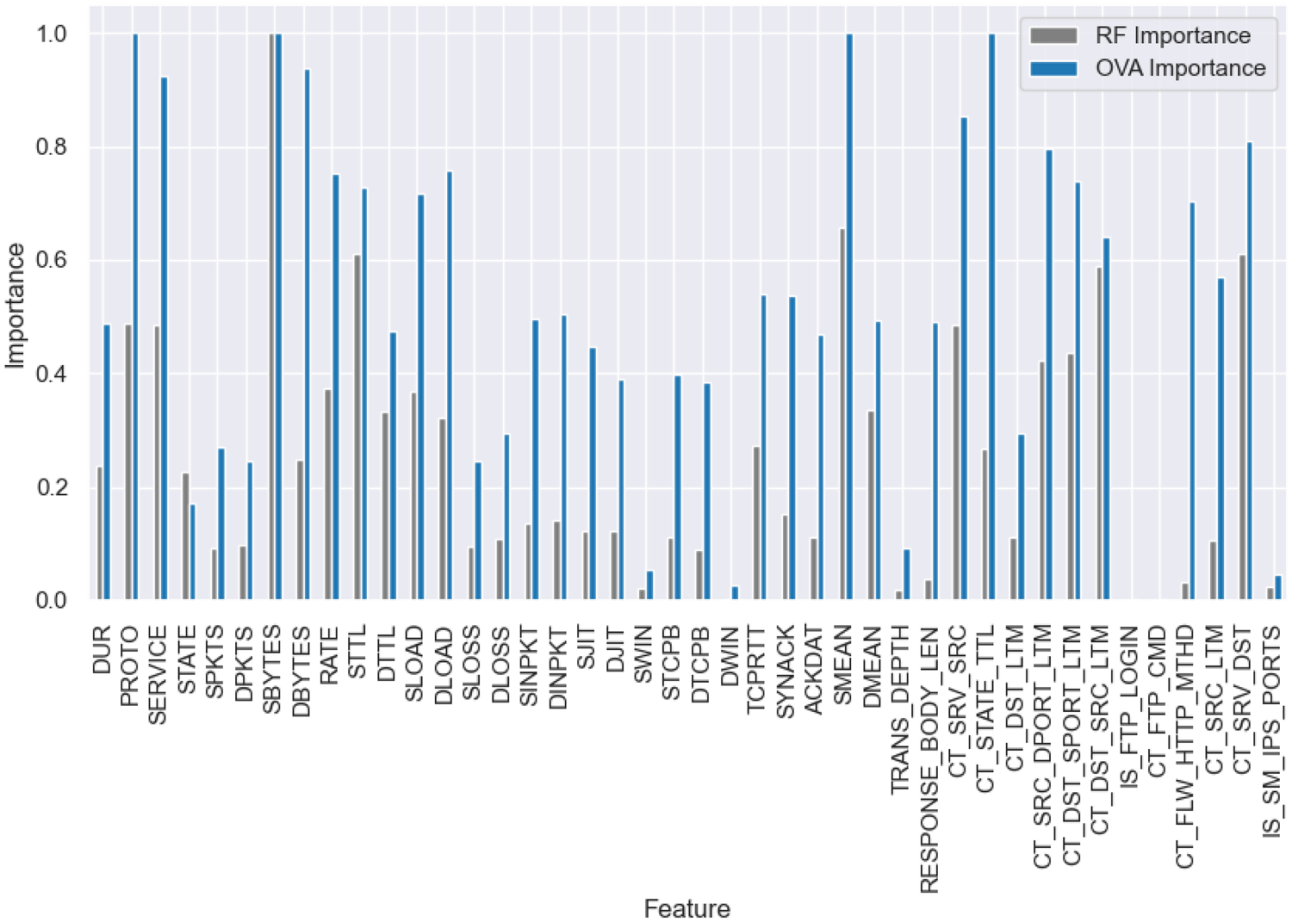
UNSW-NB15 dataset feature importance (RF vs OVA).

**Fig. 4:**
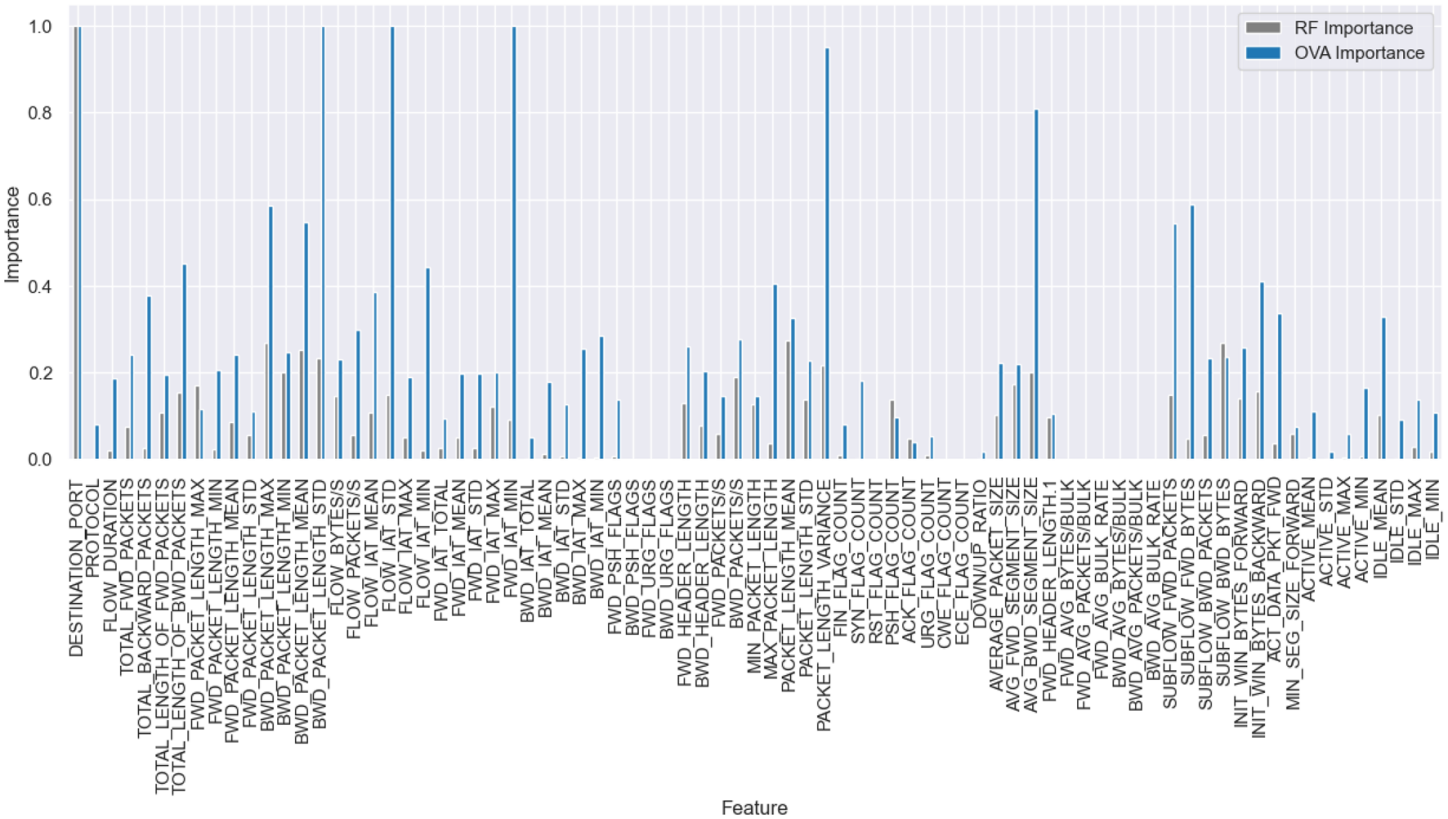
CIC-IDS2017 dataset feature importance (RF vs OVA).

Finally, we obtain the highest importance value for each feature across all classes $f_{ova}$, creating a new metric that comprehensively evaluates the importance of each feature. It’s important to note that we use the maximum value instead of the mean value, as we intend to use this importance to train an autoencoder, where these values will serve as weights for the loss generated by each feature during the training process.

6 $$\begin{aligned} f_{ova} = \max _{j} F[:,j] \quad \text {(i.e., per-feature maximum across class-wise importances)} \end{aligned}$$

Figures 2, 3, and 4 illustrate the contrast between the feature importance of random forest and our proposed feature importance approach across three different datasets. Upon examination, it becomes evident that random forest assigns low importance to certain features; however, these features are actually crucial for accurately classifying the minority classes. In our method, we assign importance to these features based on their actual significance according to all classes.

**Fig. 5:**
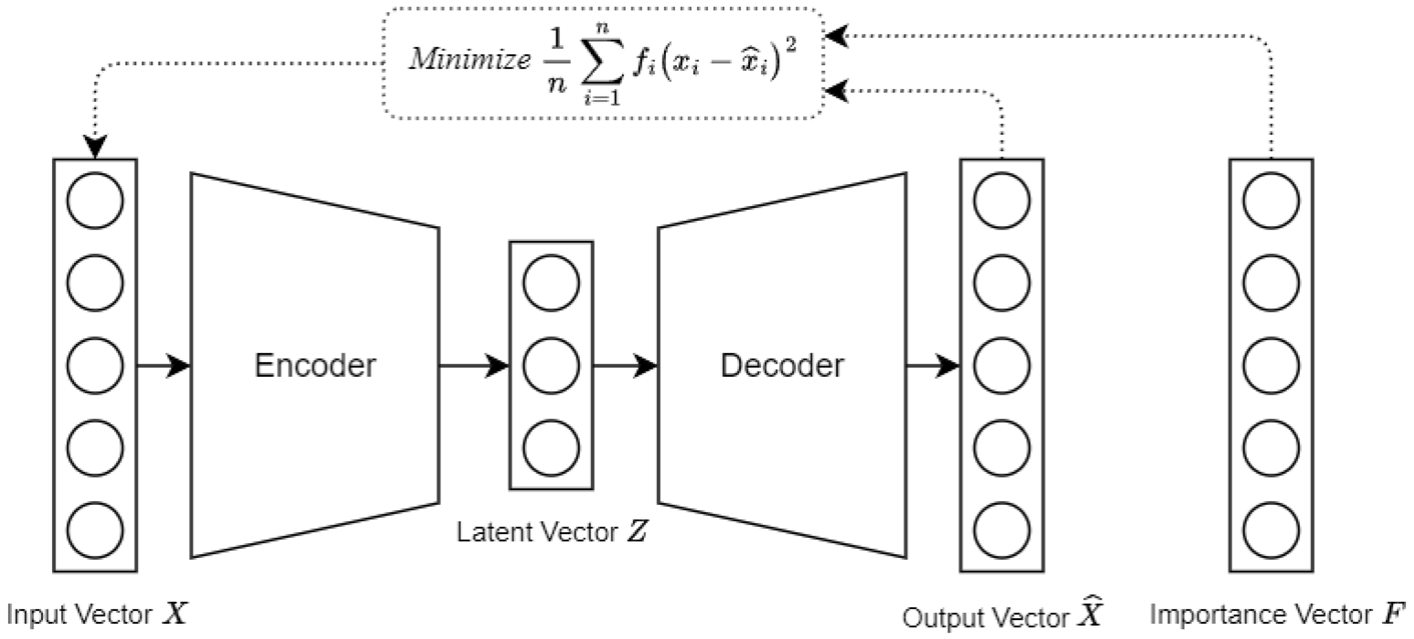
Feature importance-based autoencoder (FI-AE).

### Proposed autoencoder

An autoencoder is a type of neural network frequently employed for reducing the dimensions of data and acquiring effective representations of unlabeled data. It utilizes unsupervised learning to transform a given input *x* into a representation with fewer dimensions denoted as *h*, then subsequently reconstructs the output $\hat{x}$ from this representation to closely resemble the original input^26^. An autoencoder’s design normally has an encoder and a decoder. The encoder component compresses the input data by transforming it into a lower-dimensional representation $h = f(x)$. The encoding procedure entails the mapping of the incoming data to a hidden layer *h*. The purpose of the decoder component is to regenerate the original input data based on the latent space representation $\hat{x} = g(h)$. It performs the inverse mapping of the hidden layer *h* to the original input data *x*. During the training process, an autoencoder tries to minimize the reconstruction error by using the mean squared error loss function *MSE* to penalize *g*(*f*(*x*)) for deviating from *x*. This forces the autoencoder to capture the most significant features of the training data.

7 $$\begin{aligned} \hat{x}_i= & f(h_i) = g(f(x_i)) \end{aligned}$$

8 $$\begin{aligned} \text {MSE}= & \frac{1}{n}\sum _{i=1}^{n}(x_i - \hat{x}_i)^2 \end{aligned}$$

Autoencoders have demonstrated robust performance in dimensionality reduction tasks. However, they do not consider the importance of each feature in the input data during reconstructing the output. To incorporate the feature importance information into the autoencoder’s training process, we propose a weighted mean square error (WMSE) loss function based on our proposed OVA feature importance. The WMSE loss function assigns higher weights to losses generated from features with greater importance and lower weights to losses generated from features with lesser importance. This allows the autoencoder to prioritize important features while considering the impact of other features. Our objective is to enhance the learning process and improve the accuracy of input data reconstruction by training the autoencoder using the WMSE loss function. The formula for calculating WMSE is defined in Equation 9, where *n* represents the number of features, $f_i$ represents the importance of feature *i*, $x_i$ represents the input value, and $\hat{x}_i$ represents the predicted value.

9 $$\begin{aligned} \text {WMSE} = \frac{1}{n}\sum _{i=1}^{n} f_i (x_i - \hat{x}_i)^2 \end{aligned}$$

Our feature importance-based autoencoder (FI-AE) is structured with an encoder and a decoder as shown in Fig. 5. The encoder consists of two layers, one with 64 nodes and another with 32 nodes. The bottleneck layer has 16 nodes. The decoder, however, has two layers, one with 32 nodes and the other with 64 nodes. The LeakyReLU activation function is employed for these layers. In order to obtain stable training, we use batch normalization and dropout in the encoder/decoder (implementation details are provided in the supplementary material). We employ the Adam optimizer during training and train the model for a total of 100 epochs. During the training process, we continuously track the validation loss and save the model with the lowest validation loss for each dataset, guaranteeing that we preserve the model that performs the best. Finally, we employ the encoder component of the trained model to decrease the number of features across all benchmark datasets. The decrease in number of features enables a more compact representation of the data, which can enhance efficiency and diminish computational intricacy in later jobs or studies.

**Algorithm 1:**
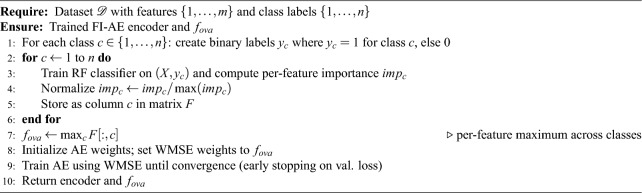
FI-AE training pipeline (pseudocode)

## Experiments and evaluation

In this section, we present the experiments conducted to evaluate the performance and effectiveness of our proposed work. We discuss the datasets used, the evaluation metrics employed, the preprocessing steps undertaken, and the results obtained by conducting these experiments and comparisons.

### Benchmark datasets

In order to examine the efficacy of our proposed model, we conducted evaluations utilizing three extensively utilized datasets in the domain. By using these datasets, we improve the efficiency of our model to make accurate predictions. The motivation for selecting these datasets is that according to^24^, 70% of the previous studies in the last 5 years have utilized these datasets. Using these well-established datasets for the proposed model can help enhance the generalization capability of the model since they have been widely employed in academic studies.

#### NSL-KDD dataset

NSL-KDD is a commonly used dataset comprising a total of 148,515 samples, each with 41 features. Among these samples, 77,206 are categorized as normal, while 71,309 are labeled as malicious. Malicious samples are further labelled as: Probing, Denial of Service (DoS), User to Root (Privilege), and Root to Local (Access) attacks^27^.

#### UNSW-NB15 dataset

UNSW-NB15 was created by the University of South Wales. It consists of 257,673 samples, each associated with 42 features. Within this dataset, 164,673 samples are classified as normal, while 93,000 samples are classified as malicious. Nine types of attacks are included in this dataset such as: Fuzzers, Backdoor, Analysis, Denial of Service (DoS), Exploits, Reconnaissance, Generic, Shellcode, and Worms^28^.

#### CIC-IDS2017 dataset

CIC-IDS2017 was established by the Canadian Institute for Cybersecurity. It comprises a substantial number of samples, totaling 2,830,541, with each sample having 79 features. Among these samples, 2,400,923 are labeled as normal, and 429,618 are labeled as malicious. Seven types of attacks are included in this dataset such as: Denial of Service (DoS), Brute force, Web Attacks, Botnet, Port scan, Heartbleed, and Infiltration^29^.

Table 2 summarizes the previously mentioned datasets.

**Table 2:** The population of normal and malicious instances, and the involved attacks in the benchmark datasets.

Dataset	Number of instances	Number of normal instances	Number of malicious instances	Involved attacks
NSL-KDD	148,515	77,206	71,309	Probing, Denial of Service (DoS), User to Root (Privilege), and Root to Local (Access)
UNSW-NB15	257,673	164,673	93,000	Fuzzers, Backdoor, Analysis, Denial of Service (DoS), Exploits, Reconnaissance, Generic, Shellcode, and Worms
CIC-IDS2017	2,830,541	2,400,923	429,618	Denial of Service (DoS), Brute force, Web Attacks, Botnet, Port scan, Heartbleed, and Infiltration

### Evaluation metrics

To assess the performance of our FI-AE model, we use standard evaluation metrics commonly used in intrusion detection systems. These metrics encompass accuracy, precision, recall, and F1-score. The following equations describe the calculation of these metrics, where *TP*, *FP*, *FN* and *TN* are true-positive, false-positive, false-negative, and true-negative respectively from the confusion matrix.

10 $$\begin{aligned} \text {Accuracy}= & \frac{TP + TN}{TP + TN + FP + FN} \end{aligned}$$

11 $$\begin{aligned} \text {Recall (R)}= & \frac{TP}{TP + FN} \end{aligned}$$

12 $$\begin{aligned} \text {Precision (P)}= & \frac{TP}{TP + FP} \end{aligned}$$

13 $$\begin{aligned} \text {F1-score}= & \frac{2 \times \text {Recall} \times \text {Precision}}{\text {Recall} + \text {Precision}} \end{aligned}$$

### Datasets preprocessing

Data preparation encompasses many crucial steps that attempt to ensure the quality and compatibility of the data. Specifically, our attention is directed towards three crucial procedures: down-sampling normal instances in the CIC-IDS2017 dataset based on clustering, scaling features using standard scaling, and encoding categorical features using target encoding.

**Fig. 6:**
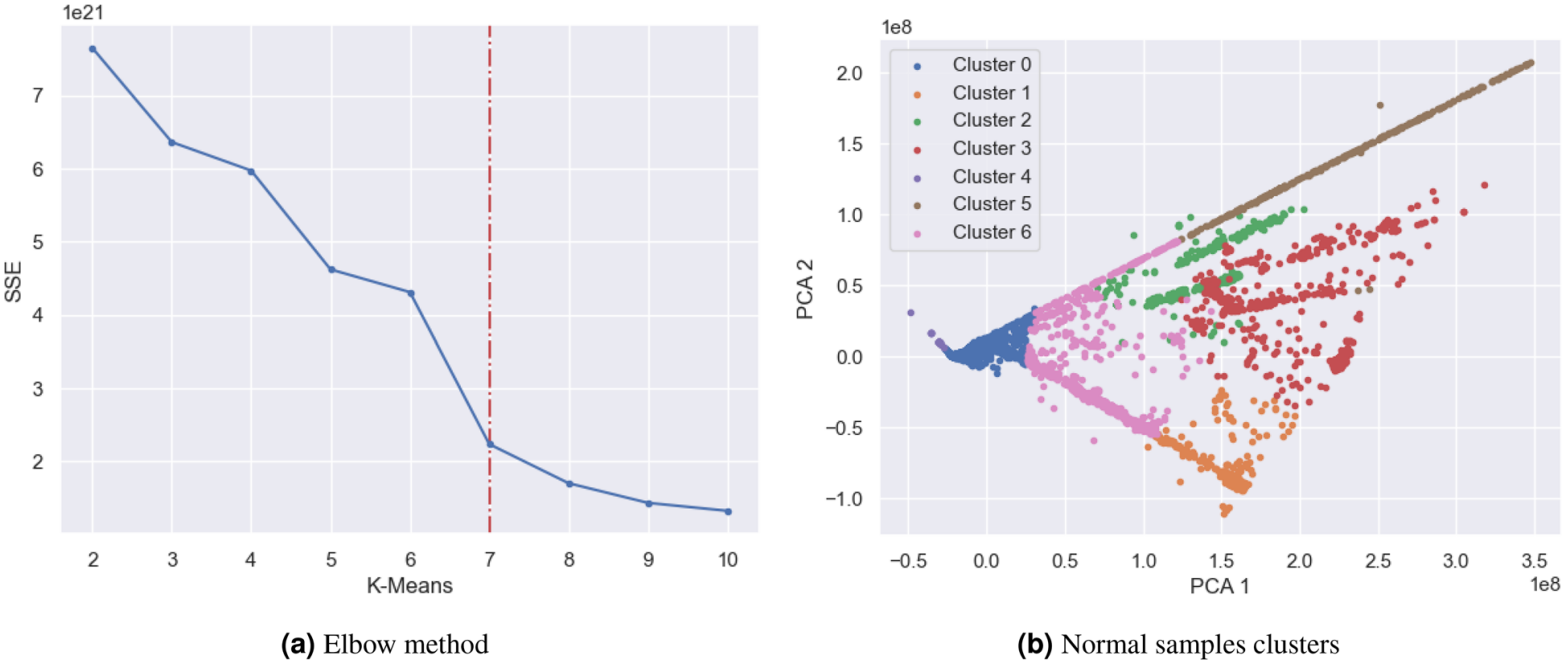
CIC-IDS2017 cluster-based downsampling for normal samples.

#### Cluster-based downsampling

Upon examining the distribution of normal attacks in the benchmark datasets, we noted the following ratios: in NSL-KDD, the ratio of normal to attack instances is 52:48; in UNSW-NB15, it is 64:36; and in CIC-IDS2017, it is 85:15. Although the ratios in the first two datasets are satisfactory, the third dataset shows a significant imbalance in the data. To address this issue, we employed a cluster centroid down-sampling technique as mentioned in^30^ specifically designed for normal samples in the CIC-IDS2017 dataset. Initially, we utilized the elbow method^31^ to determine the optimal number of clusters that would minimize the sum of squared errors (SSE) as shown in Fig. 6. This allowed us to identify the most suitable clustering configuration. We then applied the k-means algorithm to cluster the normal samples present within the CIC-IDS2017 dataset, effectively grouping together similar instances. Subsequently, we selected representative samples from each cluster to perform down-sampling of the normal samples. As a result, the total number of normal samples was reduced significantly, from an initial count of 2,400,923 samples to 360,168 samples. In contrast, the attack samples, which numbered 429,618 samples, remained unchanged and were not subject to the down-sampling process.

#### Train-test splitting

After collecting the three datasets and performing cluster-based downsampling for the normal class in the CIC-IDS2017 dataset. These datasets are split into training and testing datasets, with a ratio of 80:20. The class distribution of the resulting datasets is illustrated in Table 3 showcasing the impact of the downsampling and splitting procedures.

**Table 3:** Class distribution of the IDS datasets.

	NSL-KDD		UNSW-NB15		CIC-IDS2017	
Label	Train	Test	Train	Test	Train	Test
Normal	61,816	15,390	74,387	18,613	288,280	71,858
DoS	42,718	10,668	13,061	3,292	202,161	50,510
Probe	11,188	2,889	–	–	–	–
Access	3,002	736	–	–	–	–
Privilege	88	20	–	–	–	–
Generic	–	–	47,334	11,537	–	–
Exploits	–		35,445	9,080	–	–
Fuzzers	–	–	19,415	4,831	–	–
Reconn.	–	–	11,135	2,852	–	–
Analysis	–	–	2,158	519	–	–
Backdoor	–	–	1,861	468	–	–
Shellcode	–	–	1,202	309	–	–
Worms	–	–	140	34	–	–
Port Scan	–	–	–	–	126,988	31,942
Brute Force	–	–	–	–	11,073	2,762
Web Attack	–	–	–	–	1,720	460
Botnet	–	–	–	–	1,556	410
Infiltration	–	–	–	–	26	10
**Total**	**118,812**	**29,703**	**206,138**	**51,535**	**631,804**	**157,952**

Bold values indicate the best performance for the corresponding metric among all compared methods.

#### Features scaling and encoding

To normalize the numerical features, we employ standard scaling as depicted in Eq. 14. This process adjusts the features so that they possess a mean of zero and a variance of one, with $\mu$ representing the average value and $\sigma$ denoting the standard deviation. This technique not only hastens the training phase but also enhances the efficacy in attaining a local or global optimum.

14 $$\begin{aligned} z_i = \frac{x_i - \mu }{\sigma } \end{aligned}$$

Transforming categorical features into a numerical format is crucial for their integration into model computations. Numerous research have leveraged techniques such as one-hot encoding and label encoding to convert categorical variables. Nonetheless, label encoding falls short of accurately depicting the intrinsic value of each category by merely assigning sequential numbers ranging from 0 to the total number of categories present. These numerical assignments do not reflect the genuine significance of the categories. Conversely, one-hot encoding transforms categorical attributes into a binary matrix, correlating to the count of categories, which leads to a sparse dataset filled with zeros and a substantial expansion in feature dimensionality. In our study, we adopt target encoding^32^ as delineated in Eq. 15, which replaces a category with the average of the target variable for that category. This approach effectively addresses the limitations inherent in the aforementioned encoding strategies.

15 $$\begin{aligned} TE_i = \frac{\text {total true}(y_i)}{\text {total}(y_i)} \end{aligned}$$

**Fig. 7:**
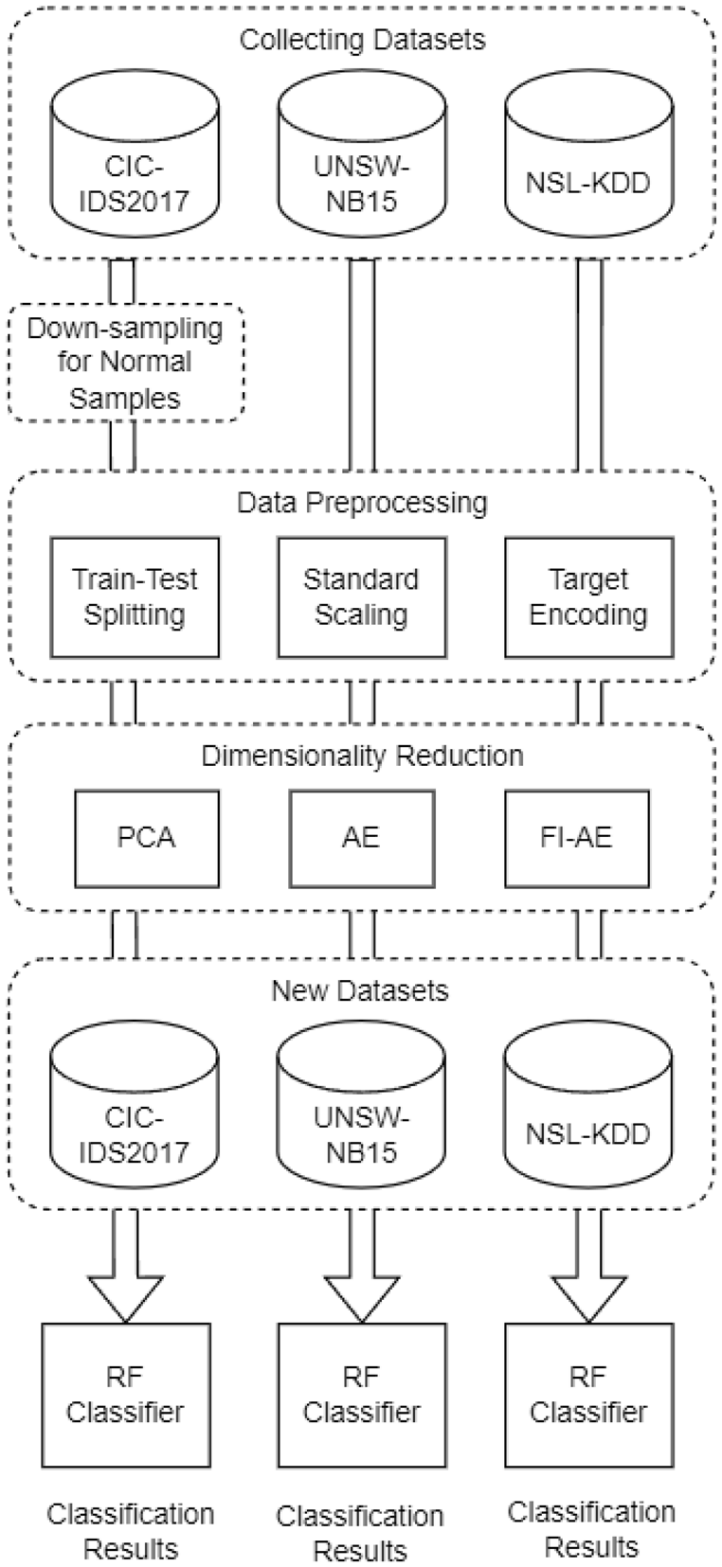
The proposed framework for dimensionality reduction.

**Fig. 8:**
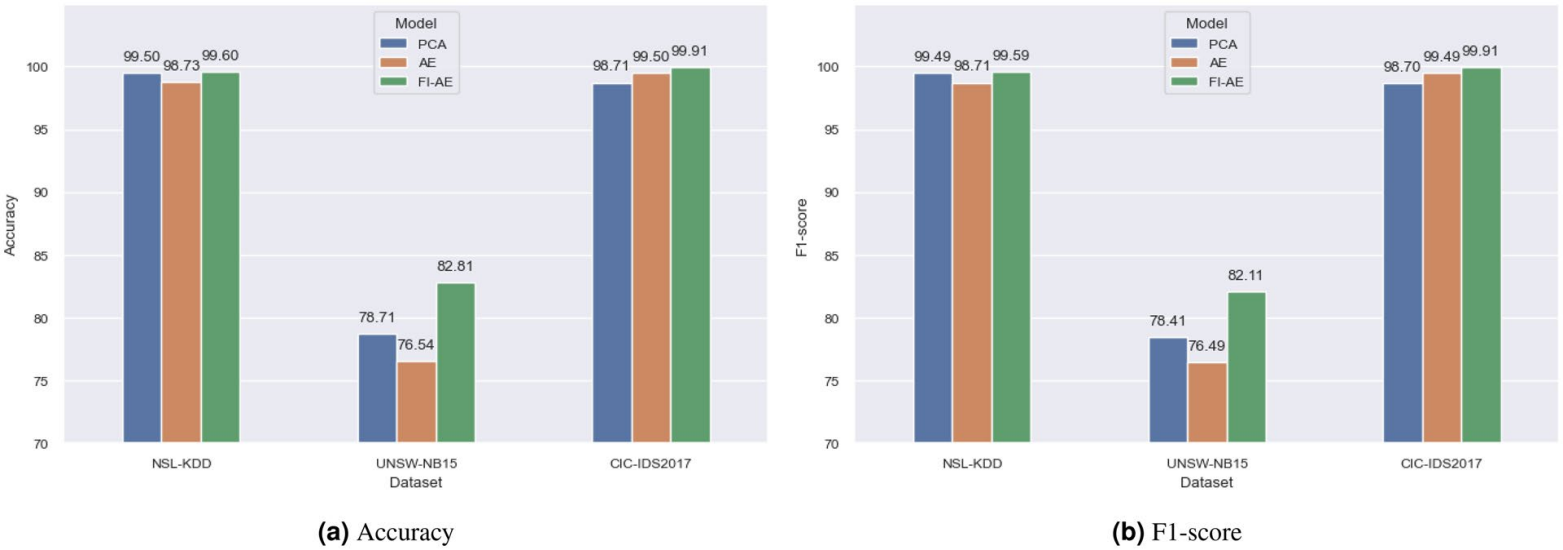
FI-AE performance comparison across the benchmark datasets.

### Results and comparison

As shown in Fig. 7, after the preprocessing stage of the benchmark datasets, we proceed with utilizing these datasets to train the autoencoder that we have created. After training the autoencoder, we employ the encoder part to decrease the dimensionality of the feature space in the benchmark datasets. Afterwards, we utilize the modified datasets, which have undergone a decrease in dimensions, to train a random forest classifier. The objective of this approach is to utilize the diminished feature space in order to enhance the classification performance of the model. We iterate through this stage employing two additional techniques: traditional autoencoder and principal component analysis (PCA).

Through a comparative analysis of the results obtained using these three techniques, we can assess their efficacy in improving the results of classification. Figure 8, and Tables 4, 5 and 6 offer a comprehensive overview of the results obtained by the three approaches when applied to the benchmark datasets, allowing us to evaluate the comparative performance and effectiveness of each approach within the context of the provided datasets.

Furthermore, to provide a comprehensive evaluation of our approach against the current state-of-the-art in dimensionality reduction for IDS, we compare the FI-AE’s performance with other related works in the literature. These comparisons are detailed for the NSL-KDD dataset in Table 7, for the UNSW-NB15 dataset in Table 8, and for the CIC-IDS2017 dataset in Table 9.

**Table 4:** Performance comparison on the NSL-KDD dataset.

	PCA			AE			FI-AE		
Attack	P	R	F1	P	R	F1	P	R	F1
Normal	99.47	99.64	99.56	98.89	99.26	99.08	99.56	99.71	99.63
DoS	99.88	99.92	99.90	99.09	99.63	99.36	99.92	99.90	99.91
Probe	99.37	99.00	99.19	97.47	94.84	96.14	99.62	99.48	99.55
Access	95.56	93.48	94.51	95.06	91.58	93.29	95.86	94.43	95.14
Privilege	75.00	60.00	66.67	66.67	30.00	41.38	85.71	60.00	70.59
Macro Avg.	93.86	90.41	91.96	91.44	83.06	85.85	96.13	90.70	**92.96**
Weighted Avg.	99.49	99.50	99.49	98.71	98.73	98.71	99.59	99.60	**99.59**
Accuracy	99.50			98.73			**99.60**		

Bold values indicate the best performance for the corresponding metric among all compared methods.

**Table 5:** Performance comparison on the UNSW-NB15 dataset.

	PCA			AE			FI-AE		
Attack	P	R	F1	P	R	F1	P	R	F1
Normal	87.63	91.04	89.30	86.24	89.18	87.69	92.30	94.44	93.36
Generic	99.65	97.53	98.58	98.98	97.43	98.20	99.73	98.08	98.89
Exploit	60.99	76.01	67.68	65.74	69.81	67.71	64.66	81.16	71.98
Fuzzers	56.79	52.08	54.34	52.85	50.48	51.64	69.90	63.07	66.31
DoS	30.73	23.09	26.37	28.49	20.90	24.11	34.95	27.86	31.00
Reconn.	84.47	70.93	77.11	78.14	75.32	76.70	92.48	74.65	82.62
Analysis	76.54	11.95	20.67	73.75	14.68	24.49	80.95	13.10	22.55
Backdoor	44.00	2.23	4.46	32.12	9.52	14.69	74.60	10.04	17.70
Shellcode	50.29	28.48	36.36	46.24	30.12	36.48	65.86	61.81	63.77
Worms	45.45	14.71	22.22	38.19	12.25	18.55	65.38	50.00	56.67
Macro Avg.	63.66	46.82	49.71	60.07	46.97	50.02	74.06	57.42	**60.49**
Weighted Avg.	78.17	78.71	78.41	76.14	76.85	76.49	82.89	82.81	**82.11**
Accuracy	78.71			76.54			**82.81**		

Bold values indicate the best performance for the corresponding metric among all compared methods.

**Table 6:** Performance comparison on the CIC-IDS2017 dataset.

	PCA			AE			FI-AE		
Attack	P	R	F1	P	R	F1	P	R	F1
Normal	97.81	99.41	98.60	99.49	99.43	99.46	99.94	99.88	99.91
DoS	99.37	97.08	98.21	99.38	99.52	99.45	99.92	99.97	99.94
Port Scan	99.85	99.95	99.90	99.83	99.93	99.88	99.87	99.96	99.92
Brute force	99.12	98.04	98.58	99.27	98.70	98.98	99.99	99.75	99.87
Web attack	97.07	93.48	95.24	99.31	93.26	96.19	99.78	97.17	98.46
Botnet	88.03	91.46	89.71	92.12	89.02	89.57	95.95	98.29	97.11
Infiltration	72.24	30.00	46.15	00.00	00.00	00.00	99.99	70.00	82.35
Macro Avg.	97.32	87.06	89.49	83.91	82.84	83.36	99.35	95.00	**96.79**
Weighted Avg.	98.72	98.71	98.70	99.49	99.50	99.49	99.91	99.91	**99.91**
Accuracy	98.71			99.50			**99.91**		

Bold values indicate the best performance for the corresponding metric among all compared methods.

At present the experiments focus on labeled benchmark datasets; the current FI-AE uses supervised per-class importances and therefore benefits from labeled attack classes at training time. Detecting previously unseen attack types (zero-day) is a distinct objective that typically requires anomaly-detection strategies (e.g., thresholding reconstruction error, one-class modeling, or density estimation on bottleneck representations). We add a dedicated discussion and recommended evaluation protocol: hold out a subset of attack classes during training and evaluate whether the bottleneck representations yield elevated reconstruction error or enable clustering/separation of unseen attacks. Future work will extend FI-AE to combine OVA weighting with semi-supervised or unsupervised anomaly detectors to improve zero-day detection.

### Computational cost and training-time considerations

- *Weighting vs model size trade-off*: WMSE introduces minimal overhead (computing per-feature weights once), while the autoencoder architecture remains compact (bottleneck 16).
- *Training-time mitigation*: use mini-batching, early stopping on validation WMSE, mixed-precision training, and learning-rate schedules.
- *Model compression*: after training, apply pruning or knowledge distillation to shrink encoder size for deployment.
- *Parallelization*: per-class RF importance computations are embarrassingly parallel and can be distributed across CPU cores.

**Table 7:** Results comparison with other related works on the NSL-KDD dataset.

Model	Precision	Recall	F1-score	Accuracy	Features
IG-PCA^14^	98.20	98.20	98.10	98.82	12
PCA-RF^33^	98.93	98.70	98.81	98.83	20
ANN^34^	83.52	92.18	87.64	97.92	25
AE-PCA^22^	92.01	75.30	82.82	82.22	30
TDTC^35^	–	98.70	–	–	**4**
STL-IDS^36^	–	–	–	99.41	–
SVM^37^	–	–	–	98.00	31
TES-IDS^38^	–	–	–	99.55	37
CANN^39^	–	99.28	–	99.46	19
FGLCC^40^	–	95.23	–	95.03	10
OS-ELM^41^	–	–	–	97.67	21
ANN^42^	–	–	–	96.89	7
**FA-AI**	**99.59**	**99.60**	**99.59**	**99.60**	16

Bold values indicate the best performance for the corresponding metric among all compared methods.

**Table 8:** Results comparison with other related works on the UNSW-NB15 dataset.

Model	Precision	Recall	F1-score	Accuracy	Features
ANN^21^	79.50	77.53	77.28	77.51	19
AE-PCA^22^	71.58	94.39	81.41	76.28	30
GRU^20^	–	–	80.84	80.84	17
TES-IDS^38^	–	–	–	81.53	19
GA-LR^43^	–	–	–	81.42	20
RF^44^	–	–	–	75.90	20
SVM^45^	–	–	–	82.11	–
**FA-AI**	**82.89**	**82.81**	**82.11**	**82.81**	16

Bold values indicate the best performance for the corresponding metric among all compared methods.

**Table 9:** Results comparison with other related works on the CIC-IDS2017 dataset.

Model	Precision	Recall	F1-score	Accuracy	Features
PCA-RF^14^	98.90	98.80	98.80	98.80	10
KF-SSA^18^	96.00	98.00	97.00	95.68	22
AE-PCA^22^	96.57	95.65	96.11	93.78	30
DOC-IDS^19^	91.10	75.60	82.60	–	–
DDR^17^	–	–	–	98.42	16
CNN^46^	–	–	–	98.61	71
XGBCON^47^	–	–	–	99.22	16
ANN^42^	–	–	–	99.77	6
**FA-AI**	**99.91**	**99.91**	**99.91**	**99.91**	16

Bold values indicate the best performance for the corresponding metric among all compared methods.

The experiments show that FI-AE provides improved downstream classification performance compared to PCA and a standard autoencoder when using a compact 16-dimensional bottleneck. The OVA importance weighting directs the autoencoder to focus on attack-discriminative features, which helps preserve minority-class signals under aggressive reduction.

### Limitations and mitigation

Important limitations include:

- *Dependency on labeled classes:* OVA requires labeled attack classes and therefore benefits from supervised information during importance computation.
- *Few-shot classes:* When attack classes have extremely few samples, RF importance estimates can be noisy.
- *Correlated features:* Importance may be spread across correlated features, reducing per-feature WMSE emphasis.

Mitigation strategies: smoothing per-class importance, grouping correlated features before importance computation, ensemble weighting, and combining with semi-supervised anomaly detectors for zero-day detection.

## Conclusion and future work

In this research, we proposed a novel autoencoder model for dimensionality reduction in intrusion detection systems called FI-AE. Our model incorporates a one-versus-all feature importance technique and a weighted mean square error loss function to address the limitations of previous approaches. Through extensive evaluations on the NSL-KDD, UNSW-NB15, and CIC-IDS2017 datasets, we demonstrated that our FI-AE model outperforms PCA and traditional AE in terms of accuracy, precision, recall, and f1-score. The results indicate that our FI-AE model effectively reduces the dimensionality of intrusion detection datasets while maintaining satisfactory performance, particularly for minority classes. The proposed feature importance technique addresses the bias of existing methods towards majority classes, ensuring a more balanced feature selection process. The use of the weighted mean square error loss function allows the autoencoder to focus on important features while still considering the contributions of other features. Overall, our FI-AE model shows great potential for enhancing the accuracy and efficiency of intrusion detection systems, contributing to the ongoing efforts to secure computer networks.

## Data Availability

The datasets used in this research are available online. NSL-KDD https://www.unb.ca/cic/datasets/nsl. UNSW-NB15 https://research.unsw.edu.au/projects/unsw-nbl5-dataset CIC-IDS2017 https://www.unb.ca/cic/datasets/ids-2017.
